# Frozen vein wrapping for chronic nerve constriction injury reduces sciatic nerve allodynia in a rat model

**DOI:** 10.1186/s12868-022-00719-7

**Published:** 2022-06-20

**Authors:** Michiaki Mukai, Kentaro Uchida, Naoya Hirosawa, Kenichi Murakami, Gen Inoue, Masayuki Miyagi, Yasuhiro Shiga, Hiroyuki Sekiguchi, Kazuhide Inage, Sumihisa Orita, Takane Suzuki, Yusuke Matsuura, Masashi Takaso, Seiji Ohtori

**Affiliations:** 1 Department of Orthopedic Surgery, Graduate School of Medicine, Chiba University, 1-8-1 Inohana, Chuo-ku, Chiba, 260-8677 Japan; 2 Department of Orthopedic Surgery, Kitasato University School of Medicine, 1-15-1 Minami-ku Kitasato, Sagamihara, Kanagawa 252-0374 Japan; 3 Department of Orthopaedic Surgery, St. Marianna University School of Medicine, 2-16-1 Sugao, Miyamae-ku, Kanagawa, 216-8511 Japan; 4 Shonan University of Medical Sciences Research Institute, Nishikubo 500, Chigasaki , Kanagawa 253-0083 Japan; 5 Department of Bioenvironmental Medicine, Graduate School of Medicine, Chiba University, 1-8-1 Inohana, Chuo-ku Chiba, 260-8677 Japan; 6 Department of Regenerative Medicine, Graduate School of Medicine, Chiba University, 1-8-1 Inohana, Chuo-ku Chiba, 260-8677 Japan

**Keywords:** Peripheral nerve injuries, Frozen vein wrapping, Allodynia

## Abstract

**Background:**

Autologous vein wrapping (VW) is used in the treatment of recurrent chronic constriction neuropathy and traumatic peripheral nerve injury. However, use of autologous veins is limited by the inability to obtain longer veins of sufficient length for larger sites. Frozen allograft tissue has several advantages, including its availability for large grafts, avoidance of donor-site morbidity, and shorter operation time. Here, we investigated the effect of frozen vein wrapping (FVW) in Wistar rats as a model of sciatic nerve injury.

**Results:**

The rats were grouped by treatment as (i) untreated after chronic constriction injury surgery (CCI; control group), (ii) treated with vein wrapping using freshly isolated vein (VW), and (iii) treated with vein wrapping using frozen vein (FVW). Mechanical allodynia was assessed with von Frey filaments on postoperative days (PODs) 1, 3, 5, 7, and 14. Gene expression of HO-1 was evaluated by quantitative polymerase chain reaction (qPCR). The response of heme oxygenase-1 gene, Hmox-1, expression to VW and FVW was assessed by RT-PCR. Both VW and FVW significantly increased withdrawal threshold levels compared to the untreated control group on POD 1, 3, and 5. Both VW and FVW also showed increased HO-1 expression compared to the CCI group.

**Conclusions:**

FVW increased the withdrawal threshold similar to VW in a rat CCI model for short periods. Frozen vein wrapping using vein allograft without donor site morbidity may be an alternative therapeutic option.

## Background

Neuropathic pain resulting from compressive neuropathy or traumatic peripheral nerve injury is a common and important medical problem. Even with proper surgery, the condition sometimes recurs and can become intractable. Autologous vein wrapping (VW) uses freshly isolated vein as a material to prevent re-adhesion with surrounding tissues, and has been used to improve recurrent symptoms due to nerve scarring in clinical settings. This procedure has improved outcomes in neuropathic pain resulting from recurrent compressive neuropathy and traumatic peripheral nerve injury [1–8]. We also reported that VW relieved pain behavior in a rat chronic constriction injury (CCI) model [9–11]. However, use of autologous VW has potential disadvantages, including donor site morbidity, increased operation time, and limited graft size.

Frozen allograft tissues such as bone, tendon, or heart valve are used to repair injured tissue in clinical settings. Allograft tissue has several advantages, such as its availability for large graft sizes, avoidance of donor-site morbidity, and shorter operation time [12]. Frozen vein wrapping using as vein allograft might therefore represent a practical therapeutic option without donor site morbidity. However, it remains unclear whether frozen vein wrapping (FVW) has a therapeutic effect, and it has not been used in clinical settings.

Previous studies proposed the mechanism by which VW relieves pain [9–11]. Heme oxygenase-1 (HO-1) is a rate-limiting enzyme which catalyzes oxidative degeneration of heme into biliverdin, carbon monoxide, and iron. Over-expression or induction of HO-1 is associated with potent anti-inflammatory and antinociceptive effects, both in vitro and in mice [13–16], and also ameliorates neuropathic pain induced by sciatic nerve injury [14, 17–20]. We previously reported that VW promotes HO-1 expression [9]. However, the question of whether FVW also promotes HO-1 remains unanswered.

Here, we investigated the effect of frozen vein wrapping on mechanical allodynia and HO-1 expression in a rat CCI model.

## Methods

### Animals

 All animal experiments were approved by the ethics committee of Chiba University (reference number: 2-196). Eight-week-old male Wistar rats (240–260 g; *n* = 115) were housed under controlled conditions in a semi-barrier housing system (12-h light/dark cycle, 21–23 °C, 45–65% humidity) and kept on a standard rodent chow diet (CRF-1; Oriental Yeast Co., Ltd., Tokyo, Japan). Rats were housed in standard cages (45 × 30 × 20 cm, two rats per cage).

### Preparation of graft materials

The abdominal portion of the vena cava was harvested from 20 donor rats, immediately immersed in phosphate-buffered saline (PBS), opened by sectioning along the longitudinal axis, and then cut into 8-mm lengths. A total of 60 vein graft materials were prepared from the venae cavae of 20 donor rats. Thirty vein sections from 10 rats were immediately applied to the CCI model. For frozen VW, the remaining 30 vein sections from 10 rats were stored in a freezer at − 80 °C for 1 week, then thawed at 37 °C for 1 h before use in the vein wrapping procedure.

### Cell viability of frozen vein

The freezing and thawing process leads to cell death in tissues via membrane damage, osmotic shock, and ice crystal formation [21, 22]. We investigated the viability of frozen veins, because viable cells could exhibit immunogenicity. Fresh veins and frozen (*n* = 6, each) veins which were thawed in culture medium at 37 °C were digested in 0.1% collagenase (Wako Pure Chemical) for 1 h at 37 °C. Vein-derived cells were cultured in a minimal essential media supplemented with fetal bovine serum for 7 days. After 7 days, attached cells in the culture dish were detached with a 0.25% trypsin/EDTA solution (BD Falcon, NJ, USA), counted using an automated cell counter (Countess™; Invitrogen Life Technologies, Carlsbad, CA, USA) and stained with trypan blue to measure cell viability.

### Creation of CCI model

90 rats were randomly assigned to three treatment groups (*n* = 30 each): a control group, a group undergoing vein wrapping with freshly isolated vein (VW group), and a group undergoing vein wrapping with frozen vein (FVW group). The control group rats underwent surgery to induce CCI (= CCI group). CCI of the sciatic nerve was induced under anesthesia with 100 mg/kg ketamine hydrochloride and 10 mg/kg xylazine hydrochloride according to a previously described method [23]. In the VW group, rats were treated with the vein wrapping procedure after the CCI surgery. Veins were then used as previously reported [9, 10]. Briefly, they were used in wrapping the ligated sciatic nerve, with the endothelial surface positioned adjacent to the epineurium of the nerve. In the FVW group, rats were treated with frozen vein using the same method as in the VW group.

### von Frey Test

Rats (*n* = 5 per group) were subjected to the von Frey test according to our previous report [9–11, 24] on postoperative days (PODs) 1, 3, 5, 7, and 14. Following randomization and 1-h acclimation to the test cage, the von Frey test was conducted by applying six von Frey filaments (3.922 mN, 5.882 mN, 9.804 mN, 13.725 mN, 19.608 mN, 39.216 mN) (Mono-filament Kit; Smith & Nephew, Germantown, WI) to the hind paw perpendicular to the plantar surface. For behavioral testing, rats are placed into the testing apparatus and allowed to habituate to the testing procedure. The area tested is the mid-plantar surface of the hind paw, which falls within the area of the tibial nerve branch in order to exclude evaluation of the sural and saphenous nerves [25, 26]. Stimulus strength was slowly increased or decreased to evaluate the withdrawal threshold. Baseline thresholds were measured 3 days prior to surgery. The Dixon nonparametric test [24] was used to analyze the data in accordance with a previous report [25].

### **HO-1 Gene (*Hmox1*) Expression Analysis**

We previously reported that VW increased HO-1 gene expression, namely *Hmox1* mRNA. In addition, consistent with the mRNA results, increased HO-1 protein levels were observed [9, 24]. Therefore, mRNA expression of Hmox1 in the sciatic nerve was examined by quantitative polymerase chain reaction (qPCR). In these experiments, an additional group of rats (*n* = 5) which did not receive CCI surgery or treatment before sciatic nerve resection (normal group) were used to evaluate relative gene expression levels following CCI. The rats (*n* = 5 per group) were euthanized with a dose of sodium pentobarbital intraperitoneally (150 mg/kg), and the right sciatic nerve was resected immediately after the animal’s death prior to (normal group), and 1, 3, 5, 7, and 14 days after wrapping. Scar tissue, veins and ligatures were removed from the resected nerves. After RNA extraction using Trizol solution (Thermo Fisher Scientific, Rockford, IL, USA) and complementary DNA (cDNA) synthesis using the SuperScript III First-Strand-Synthesis System (Thermo Fisher Scientific), we performed qPCR with PCR primers (Table 1) using the 25 µL reaction mixtures comprising 2 µL cDNA, 0.2 µM specific primer pair, and 12.5 µL SYBR Premix Ex Taq (Product no. RR820, Takara, Kyoto, Japan) under the following settings: initial denaturation at 95 °C for 1 min, 40 cycles of 95 °C for 5 s, and 60 °C for 30 s. Gene expressions were calculated by the delta-delta-method. Hmox1 mRNA expression was normalized to glyceraldehyde dehydrogenase (Gapdh) levels and values in the three treatment groups were compared.

**Table 1:** Sequences of primers used in this study

Gene	Direction	Primer sequence (5′–3′)	Product size (bp)
*Hmox1*	Sense	GAG CGA AAC AAG CAG AAC CC	167
	Antisense	ACC TCG TGG AGA CGC TTT AC	
*Gapdh*	Sense	TGC CAC TCA GAA GAC TGT GG	129
	Antisense	TTC AGC TCT GGG ATG ACC TT	

### Measurement of bFGF protein level in freeze vein

We previously reported that basic fibroblast growth factor (bFGF) stimulated HO-1 expression in sciatic nerve-derived cells [9]. To investigate the possible mechanism of *Hmox1* induction following FVW, bFGF protein levels in FVW were measured. Vein and sciatic nerve were homogenized in RIPA buffer with proteinase inhibitors. After centrifugation, supernatant was collected to measure the total protein and bFGF concentration. Total protein concentration was evaluated with the bicinchoninic acid assay. Samples having a protein concentration of 500 µg/mL were prepared and bFGF concentration was measured using a commercial bFGF ELISA kit (Biolegend, San Diego, CA).

### Statistical analyses

Effect sizes were calculated with a power analysis using an alpha of 0.05 and power of 0.80 in G*POWER3 to determine a sufficient sample size. Power analysis revealed that 15 rats were needed for the von Frey test to detect a difference between the CCI, VW and FVW groups. All statistical comparisons were conducted using SPSS (version 19.0; SPSS Inc., Chicago, IL). The normality and variances of the data was assessed with the Shapiro–Wilk and F test, respectively. As the data were normally distributed and the intergroup variances were similar, intergroup variances were similar, and two-way analysis of variance (ANOVA) followed by Tukey’s multiple comparisons test was used to compare Hmox1 mRNA levels among the control, VW, and FVW groups. The t-test was used to compare bFGF protein levels between vein and sciatic nerve. *P* < 0.05 was considered statistically significant.

## Results

### Cell viability in frozen vein

Seven days after culture, 4.3 ± 1.2 × 10^5^ cells were isolated from fresh vein. In contrast, no adherent cells were observed in frozen vein.

## von Frey tests

**Fig. 1:**
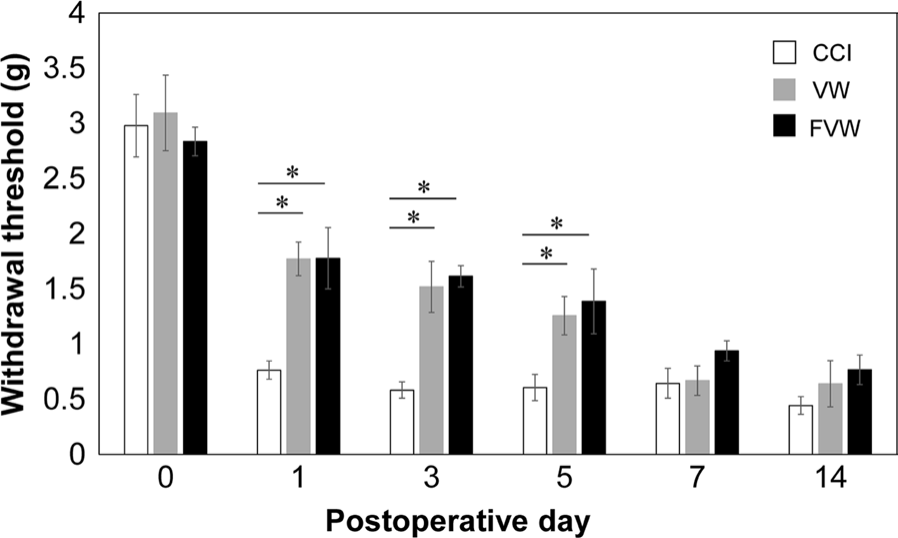
Withdrawal threshold in the CCI, vein wrapping (VW) and frozen vein wrapping (FVW) groups. Withdrawal threshold in the CCI, vein wrapping (VW) and frozen vein wrapping (FVW) groups. Data show mean ± standard error (*n* = 5, each time point). *p < 0.05

Mechanical allodynia was seen in rats in the CCI group on POD 1, and continually observed in the first 2 weeks post-surgery. Withdrawal threshold was significantly higher in the VW and FVW groups than in the CCI groups from POD 1 (*p* < 0.05; Fig. 1), POD 3 (*p* < 0.05; Fig. 1) and POD 5 (*p* < 0.05; Fig. 1). On POD 7 and 14, withdrawal threshold was higher in the VW and FVW groups than in the CCI groups, albeit that the difference was not significant. There was no significant difference between the VW and FVW groups.

### ***Hmox1*mRNA expression**

**Fig. 2:**
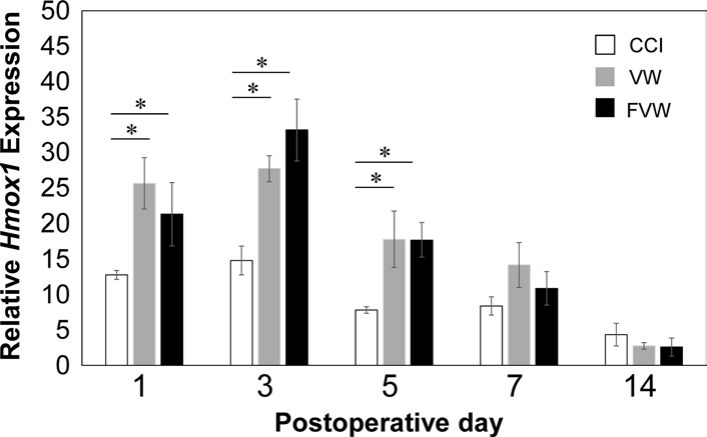
Effect of frozen vein wrapping on *Hmox1* expression. Effect of frozen vein wrapping on Hmox1 messenger RNA (mRNA) levels in sciatic nerve after chronic constriction injury (CCI). Data show mean ± standard error (*n* = 5, each time point). *p < 0.05

Hmox1 mRNA expression was significantly higher in the VW and FVW groups than in the control groups on POD 1 (p < 0.05; Fig. 2), POD 3 (p < 0.05, respectively; Fig. 2), and POD 5 (P < 0.05, respectively; Fig. 2). There was no significant difference between the VW and FVW groups.

#### bFGF concentration in vein

**Fig. 3:**
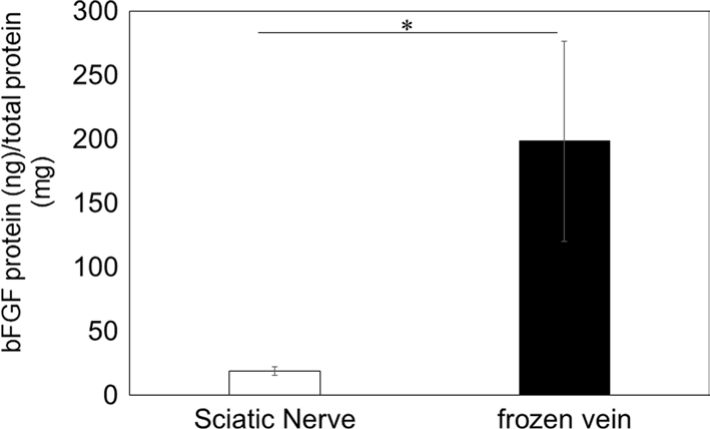
bFGF concentration in vein and sciatic nerve. bFGF protein concentration (bFGF protein (ng)/total protein (mg)) in vein and sciatic nerve. Data show mean ± standard error (*n* = 5). *p < 0.05

To investigate the possible mechanism of *Hmox1* induction following FVW, bFGF concentration was measured, and shown to be significantly higher (10.5-fold) in vein than in sciatic nerve (*p* < 0.05, Fig. 3).

## Discussion

In this study, we investigated the therapeutic effects of frozen vein wrapping for recurrent compressive neuropathy and traumatic peripheral nerve injury. Frozen vein wrapping increased the withdraw threshold level similarly to fresh vein wrapping. In addition, qPCR revealed that both fresh and frozen vein wrapping promoted *Hmox1* expression following CCI. Together, these findings suggest that frozen vein may become an alternative source of large grafts as a practical therapeutic option.

Autologous vein is generally selected for use in VW to treat recurrent chronic constriction neuropathy. However, some studies reported that glutaraldehyde-preserved allogenic vein seemed to induce a marked inflammatory response [27, 28], and that epineural scarring and adherence to the underlying nerve also had an adverse effect compared to autografting [28]. A recent study indicated that glutaraldehyde stimulated peripheral nerve scar formation and resulted in functional deficiencies [29]. In our present study, frozen VW relieved the mechanical allodynia following CCI similar to fresh VW in a rat CCI model. The freeze-thaw process markedly reduces the number of viable cells and immunogenicity in tendon cells [22]. In our study, no viable cells were observed in frozen vein. Therefore, frozen vein allograft may be a treatment option for larger sites requiring long veins.

Previous studies proposed the mechanism that autologous vein wrapping exerted its therapeutic effect through vein-derived trophic factors [9, 10, 24]. We previously reported that *Hmox1* following fresh VW and recombinant bFGF stimulate Hmox1 expression in sciatic nerve cells in vitro [9]. In our present study, even in the absence of viable cells, FVW promoted *Hmox1* expression, similarly to fresh VW. Extracellular matrix (ECM) contains growth factors which are released by proteolytic cleavage [30, 31]. bFGF is bound to heparan sulfate in the ECM and is released and activated for angiogenesis [30]. Here, frozen vein contained a higher amount of bFGF than sciatic nerve. We previously reported that bFGF stimulated rat sciatic nerve in vitro and that bFGF-absorbed collagen sheet wrapping promoted Hmox1 expression and improved mechanical allodynia [24]. Our present observation suggests that ECM-derived bFGF in vein may induce *Hmox1* expression.

VW and FVW significantly increased in mechanical withdrawal thresholds between POD1, 3 and 5. However, mechanical withdrawal thresholds were not significantly different at POD7 and 14, respectively. A previous study reported a clear and significant decrease in mechanical withdrawal threshold in a CCI model at POD1 and POD3, and that this decrease started again from POD7 and persisted for an extended period [26]. This continuous decrease in mechanical withdrawal threshold is associated with the activation of spinal microglia, which is further enhanced from POD7 or later following CCI [32]. Therefore, VW and FVW may have no efficacy in POD7 and POD14, consistent with pathology of the spinal cord.

Two limitations of this study warrant mention. First, only mechanical allodynia was used to assess pain. Evaluation of thermal and mechanical hyperalgesia, and walking track analysis were not determined. Second, we did not evaluate protein expression.

## Conclusions

FVW increased the withdrawal threshold in a similar manner to VW in a rat CCI model for short periods. Frozen vein wrapping using vein allografts without donor site morbidity may be an alternative therapeutic option.

## Data Availability

The datasets used and/or analysed during the current study are available from the corresponding author on reasonable request.

## Abbreviations

VW: Vein wrapping
FVW: Frozen vein wrapping
CCI: Chronic constriction injury
HO-1: Heme oxygenase-1
PBS: Phosphate-buffered saline
qPCR: Quantitative polymerase chain reaction

## References

[1] Campbell JT, Schon LC, Burkhardt LD (1998). Histopathologic findings in autogenous saphenous vein graft wrapping for recurrent tarsal tunnel syndrome: a case report. Foot Ankle Int.

[2] Easley ME, Schon LC (2000). Peripheral nerve vein wrapping for intractable lower extremity pain. Foot Ankle Int.

[3] Kokkalis ZT, Jain S, Sotereanos DG (2010). Vein wrapping at cubital tunnel for ulnar nerve problems. J Shoulder Elbow Surg.

[4] Mulgrew S, Kanabar GP, Papanastasiou S (2012). Further evidence for treatment of recalcitrant neuropathy of the upper limb with autologous vein wrapping. Ann Plast Surg.

[5] Sadek AF, Fouly EH, Hamdy M (2014). Functional and electrophysiological outcome after autogenous vein wrapping of primary repaired ulnar nerves. Microsurgery.

[6] Schon LC, Anderson CD, Easley ME, Lam PW, Trnka HJ, Lumsden DB (2001). Surgical treatment of chronic lower extremity neuropathic pain. Clin Orthop Relat Res.

[7] Sotereanos DG, Giannakopoulos PN, Mitsionis GI, Xu J, Herndon JH (1995). Vein-graft wrapping for the treatment of recurrent compression of the median nerve. Microsurgery.

[8] Varitimidis SE, Riano F, Sotereanos DG (2000). Recalcitrant post-surgical neuropathy of the ulnar nerve at the elbow: treatment with autogenous saphenous vein wrapping. J Reconstr Microsurg.

[9] Hirosawa N, Uchida K, Kuniyoshi K, Murakami K, Inoue G, Miyagi M (2018). Vein wrapping facilitates basic fibroblast growth factor-induced heme oxygenase-1 expression following chronic nerve constriction injury. J Orthop Res.

[10] Hirosawa N, Uchida K, Kuniyoshi K, Murakami K, Inoue G, Miyagi M (2018). Vein wrapping promotes M2 macrophage polarization in a rat chronic constriction injury model. J Orthop Res.

[11] Murakami K, Kuniyoshi K, Iwakura N, Matsuura Y, Suzuki T, Takahashi K (2014). Vein wrapping for chronic nerve constriction injury in a rat model: study showing increases in VEGF and HGF production and prevention of pain-associated behaviors and nerve damage. J Bone Joint Surg Am.

[12] Adam J, Beer TMT, Redondo Michael L, Christian David R, Brian J, Cole, Rachel M (2019). Frank Use of Allografts in Orthopaedic Surgery: Safety, Procurement, Storage, and Outcomes. Orthop J Sports Med.

[13] Devesa I, Ferrándiz ML, Terencio MC, Joosten LA, van den Berg WB, Alcaraz MJ (2005). Influence of heme oxygenase 1 modulation on the progression of murine collagen-induced arthritis. Arthritis Rheum.

[14] Hervera A, Leánez S, Negrete R, Motterlini R, Pol O (2012). Carbon monoxide reduces neuropathic pain and spinal microglial activation by inhibiting nitric oxide synthesis in mice. PLoS One.

[15] Megías J, Guillén MI, Clérigues V, Rojo AI, Cuadrado A, Castejón MA (2009). Heme oxygenase-1 induction modulates microsomal prostaglandin E synthase-1 expression and prostaglandin E(2) production in osteoarthritic chondrocytes. Biochem Pharmacol.

[16] Motterlini R, Haas B, Foresti R (2012). Emerging concepts on the anti-inflammatory actions of carbon monoxide-releasing molecules (CO-RMs). Med Gas Res.

[17] Castany S, Codony X, Zamanillo D, Merlos M, Verdú E, Boadas-Vaello P (2019). Repeated Sigma-1 Receptor Antagonist MR309 Administration Modulates Central Neuropathic Pain Development After Spinal Cord Injury in Mice. Front Pharmacol.

[18] Chen Y, Chen H, Xie K, Liu L, Li Y, Yu Y (2015). H2 Treatment Attenuated Pain Behavior and Cytokine Release Through the HO-1/CO Pathway in a Rat Model of Neuropathic Pain. Inflammation.

[19] Hervera A, Gou G, Leánez S, Pol O (2013). Effects of treatment with a carbon monoxide-releasing molecule and a heme oxygenase 1 inducer in the antinociceptive effects of morphine in different models of acute and chronic pain in mice. Psychopharmacology (Berl).

[20] Hervera A, Leánez S, Motterlini R, Pol O (2013). Treatment with carbon monoxide-releasing molecules and an HO-1 inducer enhances the effects and expression of µ-opioid receptors during neuropathic pain. Anesthesiology.

[21] Jang TH, Park SC, Yang JH, Kim JY, Seok JH, Park US (2017). Cryopreservation and its clinical applications. Integr Med Res.

[22] Suto K, Urabe K, Naruse K, Uchida K, Matsuura T, Mikuni-Takagaki Y (2012). Repeated freeze-thaw cycles reduce the survival rate of osteocytes in bone-tendon constructs without affecting the mechanical properties of tendons. Cell Tissue Bank.

[23] Bennett GJ, Xie YK (1988). A peripheral mononeuropathy in rat that produces disorders of pain sensation like those seen in man. Pain.

[24] Mukai M, Uchida K, Hirosawa N, Murakami K, Kuniyoshi K, Inoue G (2019). Wrapping With Basic Fibroblast Growth Factor-Impregnated Collagen Sheet Reduces Rat Sciatic Nerve Allodynia. J Orthop Res.

[25] Cobianchi S, de Cruz J, Navarro X (2014). Assessment of sensory thresholds and nociceptive fiber growth after sciatic nerve injury reveals the differential contribution of collateral reinnervation and nerve regeneration to neuropathic pain. Exp Neurol.

[26] Austin PJ, Wu A, Moalem-Taylor G (2012). Chronic constriction of the sciatic nerve and pain hypersensitivity testing in rats. J Vis Exp.

[27] Masear VR, Colgin S (1996). The treatment of epineural scarring with allograft vein wrapping. Hand Clin.

[28] Ruch DS, Spinner RM, Koman LA, Challa VR, O’Farrell D, Levin LS (1996). The histological effect of barrier vein wrapping of peripheral nerves. J Reconstr Microsurg.

[29] Lemke A, Penzenstadler C, Ferguson J, Lidinsky D, Hopf R, Bradl M (2017). A novel experimental rat model of peripheral nerve scarring that reliably mimics post-surgical complications and recurring adhesions. Dis Model Mech.

[30] Vlodavsky I, Fuks Z, Ishai-Michaeli R, Bashkin P, Levi E, Korner G (1991). Extracellular matrix-resident basic fibroblast growth factor: implication for the control of angiogenesis. J Cell Biochem.

[31] Vlodavsky I, Korner G, Ishai-Michaeli R, Bashkin P, Bar-Shavit R, Fuks Z (1990). Extracellular matrix-resident growth factors and enzymes: possible involvement in tumor metastasis and angiogenesis. Cancer Metastasis Rev.

[32] Nishihara T, Tanaka J, Sekiya K, Nishikawa Y, Abe N, Hamada T (2020). Chronic constriction injury of the sciatic nerve in rats causes different activation modes of microglia between the anterior and posterior horns of the spinal cord. Neurochem Int.

